# Extranuclear Estrogen Receptors Mediate the Neuroprotective Effects of Estrogen in the Rat Hippocampus

**DOI:** 10.1371/journal.pone.0009851

**Published:** 2010-05-07

**Authors:** Li-cai Yang, Quan-Guang Zhang, Cai-feng Zhou, Fang Yang, Yi-dong Zhang, Rui-min Wang, Darrell W. Brann

**Affiliations:** 1 Experimental and Research Center, North China Coal Medical University, Tangshan, Hebei, People's Republic of China; 2 Institute of Molecular Medicine and Genetics, Medical College of Georgia, Augusta, Georgia, United States of America; University of Parma, Italy

## Abstract

**Background:**

17β-estradiol (E2) has been implicated to exert neuroprotective effects in the brain following cerebral ischemia. Classically, E2 is thought to exert its effects via genomic signaling mediated by interaction with nuclear estrogen receptors. However, the role and contribution of *extranuclear* estrogen receptors (ER) is unclear and was the subject of the current study.

**Methodology/Principal Findings:**

To accomplish this goal, we employed two E2 conjugates (E2 dendrimer, EDC, and E2-BSA) that can interact with extranuclear ER and exert rapid nongenomic signaling, but lack the ability to interact with nuclear ER due to their inability to enter the nucleus. EDC or E2-BSA (10 µM) was injected icv 60 min prior to global cerebral ischemia (GCI). FITC-tagged EDC or E2-BSA revealed high uptake in the hippocampal CA1 region after icv injection, with a membrane (extranuclear) localization pattern in cells. Both EDC and E2-BSA exerted robust neuroprotection in the CA1 against GCI, and the effect was blocked by the ER antagonist, ICI182,780. EDC and E2-BSA both rapidly enhanced activation of the prosurvival kinases, ERK and Akt, while attenuating activation of the proapoptotic kinase, JNK following GCI, effects that were blocked by ICI182,780. Administration of an MEK or PI3K inhibitor blocked the neuroprotective effects of EDC and E2-BSA. Further studies showed that EDC increased p-CREB and BDNF in the CA1 region in an ERK- and Akt-dependent manner, and that cognitive outcome after GCI was preserved by EDC in an ER-dependent manner.

**Conclusions/Significance:**

In conclusion, the current study demonstrates that activation of extranuclear ER results in induction of ERK-Akt-CREB-BDNF signaling in the hippocampal CA1 region, which significantly reduces ischemic neuronal injury and preserves cognitive function following GCI. The study adds to a growing literature that suggests that extranuclear ER can have important actions in the brain.

## Introduction

17β-Estradiol (E2) has been implicated to be neuroprotective against a variety of neurodegenerative disorders, including stroke, Alzheimer's disease and Parkinson's disease, although controversy exists [1]. For instance, a number of studies have documented that women are “protected” against stroke relative to men – at least until the years of menopause, when E2 levels decline. Intriguingly, stroke *outcome* in postmenopausal women has been shown in several studies to be worse as compared to males, with postmenopausal women having a significantly higher disability and fatality rate as compared to men [2], [3], [4], [5]. While there may be many reasons for the worse stroke outcome in women, it is interesting that the onset and diminished outcome of stroke in women parallels the time period of falling E2 levels that occurs after menopause. Numerous studies have shown that *exogenous* administration of E2 dramatically reduces infarct volume following focal or global cerebral ischemia in ovariectomized female mice, rats and gerbils, and in male rats and gerbils [1], [6], [7], [8], [9], [10]. Two estrogen receptor (ER) isoforms have been identified to date, ERα and ERβ, both of which are expressed in the adult brain and thus could mediate the neuroprotection by E2 [1], [11], [12]. ERα has been implicated as particularly important in the neuroprotective effects of E2, as evidenced by the fact that E2-mediated neuroprotection against middle cerebral artery occlusion (MCAO)-induced cerebral ischemia is lost in OVX ERα knockout mice, but not ERβ-KO mice [13], [14], and by the fact that ERα, but not ERβ, antisense oligonucleotides significantly attenuate E2 neuroprotection in the hippocampal CA1 region following global cerebral ischemia (GCI) [15]. However, use of purported selective ERα and ERβ agonists in the GCI model, suggested that both ER subtypes may contribute to E2 neuroprotection in the hippocampal CA1 region of the brain [16].

It has been predominantly thought that E2 neuroprotection in the brain is mediated principally by the “classical” nuclear ER-mediated genomic signaling pathway, which involves E2 interaction with nuclear ER and regulation of transcription of various genes that mediate neuroprotection. For instance, E2 has been shown to increase the expression of the anti-apoptotic gene, *bcl-2*, in the ischemic penumbra following MCAO and global ischemia [17]. E2 also increases *bcl-2 in vitro* in rat hippocampal neurons and human NT2 neurons [18], [19], while it inhibits expression of pro-apoptotic BAD (bcl-2-antagonist of cell death) [17], [18], [19], [20]. Additionally, E2 enhances expression of the antiapoptotic prosurvival factor, survivin in the hippocampus CA1 following GCI, which facilitates neuronal survival [6]. E2 has also been shown to enhance expression of brain derived neurotrophic factor (BDNF) in the brain, which has been implicated as a neuroprotective factor and to be important for synaptic plasticity, learning, and memory [21], [22].

In addition to genomic signaling, there is increasing evidence that rapid nongenomic signaling via membrane localized extranuclear ER may also play a role in mediating E2 neuroprotective effects in the brain [23], [24], [25]. Along these lines, several laboratories have shown that the rapid activation of extracellular signal-regulated kinases 1,2 (ERKs) by E2 is critical for its neuroprotective effects, as administration of a MEK inhibitor blocks E2 neuroprotection in neurons in vitro [24], [25], [26]. Furthermore, E2-induces ERK activation in the CA1 region after GCI, which is critical for its neuroprotective effects as treatment with a MEK inhibitor blocked E2-induced ERK activation and E2 neuroprotection in the hippocampus [27] Likewise, a role for the prosurvival serine kinase Akt in E2 neuroprotection has been implicated, as E2 rapidly up-regulates Akt activation in cortical neurons *in vitro* [28], and in the hippocampus CA1 *in vivo* following GCI [29], while treatment with a PI3K inhibitor attenuates the neuroprotective effects of E2 both *in vitro* and *in vivo* [28], [29]. In addition, we recently demonstrated that E2 attenuates the rapid activation of the proapoptotic signaling kinase, JNK in the hippocampal CA1 region after GCI [6]. As a whole, these findings suggest that E2-induced rapid nongenomic signaling may play a critical role in E2 neuroprotection.

However, since the above studies principally used E2, which can activate *both* extranuclear and nuclear estrogen receptors, it has been difficult to distinguish the importance and contribution of extranuclear receptor-mediated signaling in E2 neuroprotective effects. To address this issue, the current study employed two E2 conjugates, E2-BSA conjugate [30], [31], [32] and the newer E2 dendrimer conjugate (EDC) [33], which due to their size and charge cannot enter the cell nucleus. EDC and E2-BSA retain their ability to induce rapid extranuclear-mediated nongenomic signaling, but lack significant nuclear ER-mediated genomic signaling ability due to their inability to enter the cell nucleus and interact with nuclear ER [32], [33]. Thus, their use has the potential to provide important insight into the role and importance of extranuclear estrogen receptors in E2 neuroprotective effects in cerebral ischemia. The results of our study reveal that EDC and E2-BSA administered intracerebroventrically (icv) rapidly activates ERK, Akt and CREB signaling pathways in the hippocampus, enhances levels of the CREB transcriptional target, brain-derived neurotrophic factor (BDNF), strongly protects the hippocampal CA1 region against neuronal cell death, and significantly improves hippocampal-dependent cognitive function in the Morris water maze following GCI. The study thus provides important new evidence of a critical role for *extranuclear* estrogen receptor activation in estrogen-induced neuroprotection and improved functional cognitive outcome following GCI, and suggests that ERK-Akt-CREB-BDNF signaling is an important component mediating extranuclear estrogen receptor beneficial neural effects.

## Materials and Methods

### Materials

Rabbit polyclonal anti-p-Akt1 (Ser47, sc-7985-R), anti-p-JNK (Thr183/Tyr185, sc-12882-R), anti-JNK (sc-572), anti-Actin (sc-10731), goat polyclonal anti-Akt (sc-7126), and anti-ERK (sc-94-G) antibodies were acquired from Santa Cruz Biotechnology (Santa Cruz, CA, USA). Rabbit anti-p-ERK1/2 (pTpY185/187, 44-680G) was from Biosource (Camarillo, CA, USA). Mouse monoclonal anti-NeuN (MAB377) was acquired from Millipore (Billerica, MA, USA). Rabbit p-CREB (Ser133, #9189) and mouse CREB (#9104) were from Cell Signaling Technology (Beverly, MA, USA). The secondary antibodies used were goat anti-rabbit IgG, goat anti-mouse IgG, and donkey anti-goat IgG and were from Sigma Chemical Co. (St. Louis, MO, USA). Dimethylsulfoxide (DMSO) was also from Sigma. Alexa Fluor 594 donkey anti-mouse IgG (A21203), Alexa Fluor 488 donkey anti-rabbit IgG (A21206) and Alexa Fluor 488 donkey anti-goat IgG (A11055) were purchased from Invitrogen (Carlsbad, CA, USA). PD98059, LY 294,002 and ICI 182,780 were purchased from Tocris Cookson, Inc (St. Louis, MO, USA). NBT/BCIP was obtained from Promega Corporation (Madison, WI, USA).

### Methods

#### Global cerebral ischemia

Adult Sprague Dawley female rats weighting 250-300g were used throughout the study. All procedures were approved by the Medical College of Georgia institutional committee for care and use of animals and were in accordance with National Institutes of Health guidelines. The rats were bilaterally ovariectomized under anesthesia, and one week later four-vessel occlusion (4-VO) cerebral ischemia was carried out. Briefly, under anesthesia with chloral hydrate (350 mg/kg, i.p.), vertebral arteries were permanently electrocauterized with a monopolar coagulator through the alar foramens of the first cervical vertebra. Common carotid arteries were exposed through the ventral midline cervical incision and ligatures were placed loosely around each artery without interrupting the carotid blood flow. On the next day, under light halothane anesthesia, the common carotid arteries were re-exposed and occluded with aneurysm clips to induce forebrain ischemia. After 10 min ischemia, blood flow was restored by releasing the clips. Criteria for forebrain ischemia were: completely flat electroencephalographs, maintenance of dilated pupils, absence of a cornea reflex when exposed to strong light stimulation, and maintenance of rectal temperature at about 37°C. Those not matching the criteria or with seizures were excluded. Sham animals received the same surgical procedures except bilateral carotid arteries were not occluded.

#### Morris water maze test

The spatial learning and memory of the rats were evaluated by Morris water maze as described previously [34], [35]. The maze consisted of black circular pool (diameter 2.14 m, height 80 cm, filled with water at 21–22°C to a height of 50 cm). The pool was divided into four zones by the software with a hidden platform (9 cm in diameter, 1.5–2.0 cm below the water line) placed in one of the zones. The rat was placed in the water facing the wall at one random start location of four (north, south, east and west, locating at equal distances from each other on the pool rim). Each rat was allowed to find the submerged platform within 90s, and rest on it for 20s. If the rat failed to find the hidden platform within 90s, it was placed on the platform for 20s. The procedure was repeated for all the four start locations. The latency time, representing the average of the four trails, to reach the platform and distance were recorded. Two sessions of four trails were conducted on the first testing day, and the interval was 4h. The first session was considered as training procedure. The second was formal testing and was conducted daily on the next 2 days. Four hours after the last trail, a probe trail was performed within 90s in which the platform was removed from the tank. The rat was placed in the water at the same random start location, and time spent in the quadrant of the pool, which previously contained the platform were used to assess performance of learning and memory of the rats.

#### Sample preparation

The rats were sacrificed under anesthesia at 10, 30min, 3h, 6h or 1 day after ischemia. The hippocampal CA1 region was micro-dissected from both sides of the hippocampal fissure at 0°C and quickly frozen in liquid nitrogen. Tissues were homogenized in a 1∶10 (w/v) ice-cold homogenization buffer for 10 min consisting of 50 MOPS (mM, PH 7.4), 150 NaCl, 20 β-glycerophosphate, 3 DTT, 2 Na_3_VO_4_, 1 EGTA, 1 EDTA, 1 NaF, 1% Triton X-100, 1% NP-40 and inhibitors of proteases and enzymes (0.5 mM PMSF, 10 µg/ml each of aprotinin, leupeptin, and pepstatin A). This was followed by centrifugation at 15,000×g for 15 minutes. The supernatant was removed and stored at −80°C until use. The protein concentrations were determined using a BCA protein assay kit with bovine serum albumin (BSA) as standard.

#### Western blotting

100 µg protein of each sample was heated at 100°C for 5min with loading buffer containing 0.125 M Tris-HCL (PH 6.8), 20% glycerol, 4% SDS, 10% mercaptoethanol and 0.002% bromphenol blue, then separated by sodium dodecyl sulfate-polyacrylamide gel electrophoresis (SDS-PAGE) using 10% acrylamide gels. The proteins were transferred onto PVDF membranes (pore size, 0.45 µm). Blotting membranes were incubated with 3% BSA in TBST (10 mmol/L Tris (PH 7.5), 150 mmol/L NaCl, 0.05% Tween-20) and probed with corresponding primary antibodies at 4°C overnight. Use alkaline phosphatase conjugated monkey anti-goat IgG or goat anti-rabbit or rabbit anti-mouse IgG as secondary antibodies and BCIP/NBT as color substrate. The bands on the membranes were scanned and analyzed with an image analyzer (Labworks Software, UVP Upland, CA, USA). Phospho-protein signals were expressed as a ratio to the corresponding total protein, and the total proteins were expressed relative to actin in the same sample. Normalized means were then expressed relative to the ratio for sham-treated animals.

#### Histochemical analysis of neuroprotection

After 7 days of reperfusion period, the animals were deeply anesthetized with chloral hydrate and transcardially perfused with 0.9% saline, followed by 4% paraformaldehyde in 0.1 M phosphate buffer (PB, pH 7.4). Brains were post-fixed in the same fixative at 4°C for 12h, cryoprotected in 30% sucrose in PB and then were cut longitudinally into 25µm sections with a cryostat. NeuN staining was performed as described previously by our laboratory [6], [15]. Briefly, sections were washed with 0.1% PBS-Triton X-100 for 3×5min and permeabilized with 0.4% Triton X-100 in PBS for 10min. After incubation with blocking solutions containing 10% normal donkey serum for 1h at room temperature in PBS containing 0.1% Triton X-100, sections were incubated in primary antibodies (mouse anti-NeuN, 1∶200) for 48h at 4°C. Sections were washed for 3×10 min, followed by incubation with Alexa Fluor 594 donkey anti-mouse antibody (1∶200) for 1h at room temperature. Sections were then washed for 4×5min with PBS-Triton X-100, then PBS, and finally with water, and mounted using water-based mounting medium. Images were captured on a Confocal Laser Scanning Microscope (Olympus FV1000). The number of NeuN-positive CA1 neurons per 1 mm length of the medial CA1 pyramidal cell layer was counted bilaterally in five sections per animal. Cell counts from the right and left hippocampus on each of the five sections were averaged to provide the mean value. A mean±SD was calculated from the data in each group and statistical analysis performed as described below.

#### Confocal Microscopy

Coronal sections were permeabilized using 0.4% Triton X-100-PBS for 10 min. Sections were then incubated for 30 min in 10% donkey serum, followed by overnight incubation at 4°C with primary antibodies - mouse anti-NeuN (1∶200) and rabbit anti-p-Akt (1∶50), or mouse anti-NeuN (1∶200) and rabbit anti-p-ERK1/2 (1∶50), or mouse anti-NeuN (1∶200) and goat anti-p-JNK. Sections were then washed for 30 min in 0.1% Triton X-100-PBS and incubated with different secondary antibodies (Alexa Fluor 594 donkey anti-mouse IgG and Alexa Fluor 488 donkey anti-rabbit IgG or Alexa Fluor 488 donkey anti-goat IgG) at room temperature for 1 h, followed by a final wash for 30 min in PBS. Sections were placed on slides and covered with mounting medium containing antifading agents (Biomeda; Thermo Fischer Scientific). Images were obtained using an Olympus FV1000 laser scanning confocal microscope.

#### Administration of drugs

EDC (10 µM in 5 µl saline), E2-BSA (10 µM in 5 µl saline), or vehicle were bilaterally infused into the lateral ventricles (from the bregma: anteroposterior, ±0.8 mm; lateral, 1.5 mm; depth, 3.5 mm) 60min before induction of GCI. The estrogen receptor antagonist ICI182,780 (50 µg), the MEK and PI3K inhibitors PD98059 (10 µg), LY294002 (25 µg) in 5 µl 1% DMSO respectively were administered icv 10min prior to EDC or E2-BSA treatment. A volume of 1µl each was infused over 5 min. Following injection, the needle was left in situ for 5min. The same volume of 1% DMSO or saline served as vehicle control.

#### Statistical Analysis

Statistical analysis of the results was carried out by one-way analysis of variance (ANOVA), followed by the least significant (LSD) or Student-Newman-Keuls tests. In all cases, *p*<0.05 was considered significant. Data was expressed as mean±SD.

## Results

### Extranuclear/membrane localization of EDC and E2-BSA in hippocampal CA1 cells after *icv* injection and enhanced neuronal survival and cognitive outcome in EDC- and E2-BSA-treated rats following global cerebral ischemia (GCI)

**Figure 1:**
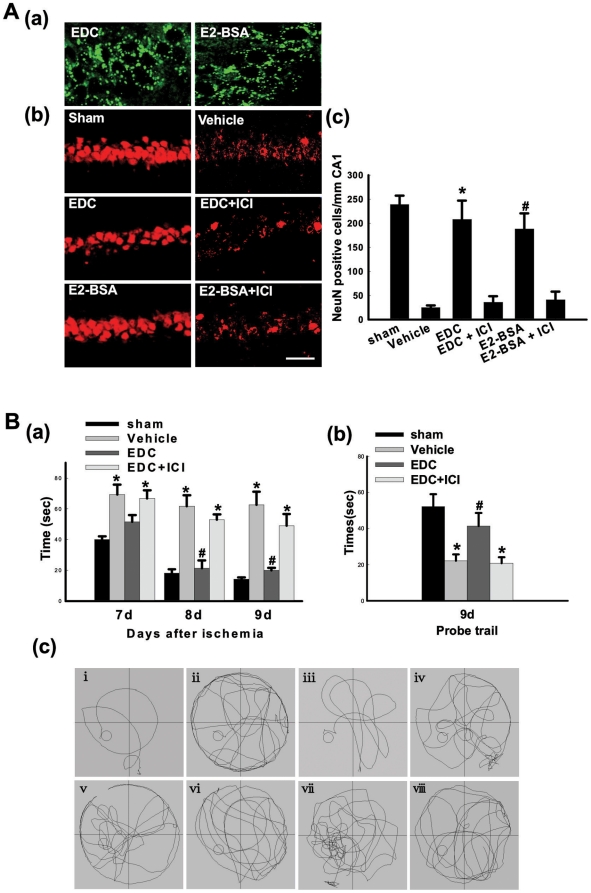
(A-a) Extranuclear localization of EDC and E2-BSA in neurons in hippocampal CA1 region of the rats. FITC-tagged EDC and E2-BSA were injected into the lateral ventricles, and 1h later, the rats were underwent transcardial perfusion and cut into 25µm coronal brain sections with a cryostat. Confocal analysis showed that EDC and E2-BSA entered neurons in the hippocampal CA1 region but are incapable of penetrating into the nucleus. **(A-b,c) EDC and E2-BSA2 protect neurons of the hippocampus CA1 region from injury induced by GCI**. NeuN immunostaining of representative hippocampus sections from sham, vehicle, EDC, EDC+ICI, E2-BSA and E2-BSA+ICI-treated female ovariectomized rats subjected to 10min GCI followed by 7d reperfusion. Global ischemia induced significant neuronal cell loss in the CA1 pyramidal cell layer. EDC or E2-BSA treatment afforded nearly complete protection from global cerebral ischemia-induced neuronal cell loss. ICI182,780 (ICI) abrogated the neuroprotection induced by EDC or E2-BSA. NeuN-positive neurons in per 1 mm length of CA1 region was counted as surviving neurons. F(5,30) = 105,^*^ *p*<0.001 vs. vehicle and EDC+ICI groups; F(5,30) = 105, ^#^ *p*<0.001 vs. vehicle and E2-BSA+ICI groups. Magnification 40×; Scale bar 50µm. **(B) Effects of EDC on spatial learning and memory ability in the Morris water maze**. (a) Latency to find the submerged platform. F(11,48) = 77,^*^ *p*<0.001 vs. sham; F(11,48) = 77, ^#^ *p*<0.001 vs. Vehicle and EDC+ICI. (b) Time spent in the quadrant, which initially contains the platform. F(3,16) = 37, ^*^ *p*<0.001 vs. sham; F(3, 16) = 37, ^#^ *p*<0.01 vs. Vehicle and EDC+ICI. (c) Representative sample paths from the maze trials (i–iv) and the probe trials (v–viii) at 9 days after reperfusion. (i,v: sham; ii,vi: vehicle; iii,vii: EDC; iv,viii: EDC+ICI). R: reperfusion. ICI: ICI182,780.

**Figure 1A** shows localization of FITC-tagged EDC and E2-BSA conjugates in the hippocampal CA1 region at 60 min following icv injection in the lateral ventricle of ovariectomized female rats. As shown in **Fig.1A (a)**, FITC-EDC and FITC-E2-BSA conjugates are heavily localized in the CA1 after icv injection, displaying a punctuate extranuclear/membrane pattern of localization. **Figure 1A (b&c)** shows the neuroprotective ability of EDC and E2-BSA administered icv 60 min prior to GCI. Examination of NeuN-positive cells at 7d after GCI revealed that Vehicle-treated animals that underwent 10-min GCI displayed a significant loss of NeuN-positive cells in the hippocampal CA1 region as compared to Sham controls. Intriguingly, the cell impermeable E2 conjugates, EDC and E2-BSA both exerted robust neuroprotection against GCI, an effect that appeared to be mediated by ER, as pretreatment with the ER antagonist, ICI182,780 completely abolished the neuroprotective effect of both EDC and E2-BSA. We next examined the effect of EDC upon functional outcome following GCI by examining EDC effect upon spatial learning and memory in rats 7–9 days after reperfusion using the Morris water maze (**Figure 1B, a–c**). As shown in **Fig 1B (a)**, Vehicle-treated animals that underwent GCI showed significant higher latencies in finding the submerged platform on days 7–9 post stroke as compared to sham control rats. In contrast, EDC-treated rats had significantly decreased latencies to find the submerged platform on day 7–9 as compared to the Vehicle group, an effect that was significantly reversed by the ER antagonist ICI182,780. Furthermore, **Fig. 1B (b)** shows that Vehicle-treated animals spent significantly less time in the quadrant where the submerged platform was located as compared to sham animals on Day 9. In contrast, EDC-treated rats spent significantly greater amount of time in the quadrant where the submerged platform was located as compared to Vehicle group, and this effect was significantly reversed by the ER antagonist, ICI182,780, suggesting ER mediation of the cognitive enhancing/preservation effect of EDC. Finally, representative sample paths for the various groups and treatments for the maze and probe trials at 9 days reperfusion are provided in **Fig. 1B (c)**, which illustrates that EDC-treated animals in the probe trials spent significantly more time in the quadrant where the platform was located.

### EDC and E2-BSA neuroprotective effects against GCI are mediated by ERK and Akt signaling pathways

#### p-Akt

**Figure 2:**
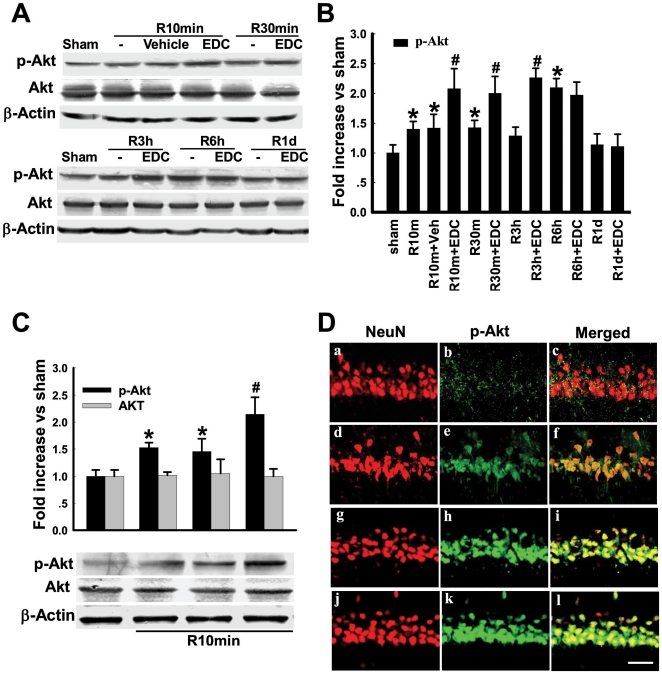
Effects of EDC and E2-BSA on activation of Akt following cerebral ischemia in the hippocampal CA1 region. (**A**) Time courses of p-Akt and Akt, as well as the effects of EDC. Rats were subjected to 10min ischemia followed by 10min, 30min, 3h, 6h and 1d reperfusion. Bands corresponding to p-Akt were scanned and the intensities represented as values were shown in (**B**). F(11,36) = 19, ^*^ *p*<0.05 vs. sham; F(11,36) = 19, ^#^ *p*<0.05, significant difference vs. Vehicle. (**C**) Effect of E2-BSA on Akt activation at 10min of reperfusion. F(3,12) = 20, ^*^ *p*<0.01 vs. sham; F(3,12) = 20, ^#^ *p*<0.05 significant difference vs. Vehicle. (**D**) Confocal analysis of NeuN and p-Akt immunostaining in hippocampus CA1 at 10min reperfusion after GCI. Sham(a,b,c); vehicle(d,e,f); R10min+EDC(g,h,i); R10min+E2-BSA(j,k,l). Magnification 40×; Scale bar 50µm.

We next examined the extranuclear/membrane signaling pathways regulated by EDC and E2-BSA, with a focus on two known pro-survival kinases, Akt and ERK, and a pro-death kinase, JNK. As shown in **Fig. 2A–B**, a time course study revealed that p-Akt levels in the CA1 increased slightly but significantly from 10min - 6h following reperfusion, with a fall back to sham levels at 1d reperfusion. Total Akt and β-actin levels did not change at any time point. EDC treatment led to a significant elevation of p-Akt levels in the CA1 from 10min - 3h, with a fall back to sham levels at 1d. **Fig. 2C** shows that E2-BSA similarly enhances p-Akt levels at 10 min after reperfusion following GCI, while total Akt levels were unchanged. **Fig. 2D**, shows representative double immunohistochemistry results for NeuN and p-Akt in the CA1 region at 10 min reperfusion after GCI. The results mirror the Western blot results observed in Fig 2A–C, with 10 min reperfusion increasing p-Akt levels slightly, with a more robust increase of p-Akt staining observed following EDC and E2-BSA treatment. Note also the p-Akt induction is colocalized with NeuN, suggesting that the elevation of p-Akt by EDC and E2-BSA occurs in neurons in the hippocampal CA1 region.

#### p-ERK

**Figure 3:**
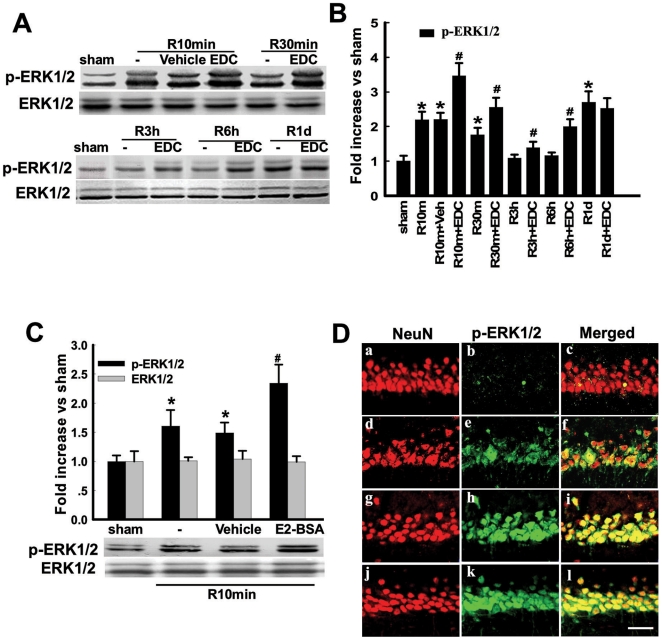
Effects of EDC and E2-BSA on phosphorylation level and protein expression of ERK1/2 following cerebral ischemia in the hippocampal CA1 region. The time-course of p-ERK1/2 and the effect of EDC were shown in **Fig. 3A**. Rats were subjected to 10min ischemia followed by 10min, 30min, 3h, 6h and 1d reperfusion. (**B**) Bands corresponding to p-ERK1/2 were scanned and the intensities represented as values. F(11,36) = 41, ^*^ *p*<0.001 vs. sham; F(11,36) = 41, ^#^ *p*<0.05 vs. Vehicle. (**C**) Effect of E2-BSA on p-ERK1/2 at 10min of reperfusion. F(3,12) = 22, ^*^ *p*<0.05 vs. sham; F(3,12) = 22, ^#^ *p*<0.001 vs. Vehicle. (**D**) Confocal analysis of NeuN and p-ERK1/2 immunostaining in hippocampus CA1 at 10min reperfusion after global cerebral ischemia. Sham(a,b,c); vehicle(d,e,f); R10min+EDC(g,h,i); R10min+E2-BSA(j,k,l). Magnification 40×; Scale bar 50µm.

**Fig. 3A–D** shows the effect of EDC and E2-BSA upon p-ERK levels in the CA1 region after GCI. As shown in **Fig. 3A–B**, reperfusion had a biphasic elevation of p-ERK levels in the CA1 after GCI, with an initial 2-fold increase over sham observed at 10min, followed by a fall back to sham levels from 3h–6h, and a second elevation of p-ERK at 1d. It should be noted that p-ERK1 and p-ERK2 changed in parallel in our study, and that total ERK levels showed no change at any timepoint. EDC treatment significant increased p-ERK levels in the CA1 region from 10 min - 6h, as compared to the reperfusion or vehicle control. **Fig. 3C** shows that E2-BSA has a similar enhancing effect upon p-ERK levels in the CA1 region at 10min reperfusion following GCI. Confocal analysis of double immunohistochemistry for NeuN and p-ERK confirmed the Western blot results, demonstrating that EDC and E2-BSA markedly enhance p-ERK levels in the CA1 at 10min reperfusion after GCI, with the increase in p-ERK occurring in CA1 neurons (**Fig. 3D**).

#### p-JNK

**Figure 4:**
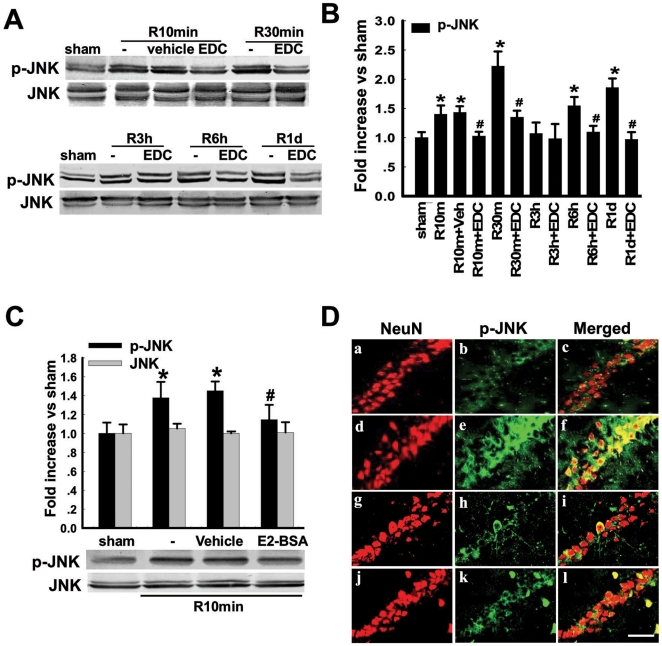
Effects of EDC and E2-BSA on phosphorylation level of JNK following cerebral ischemia in the hippocampal CA1 region. (**A**) Time courses of p-JNK and JNK, as well as the effects of EDC. Rats were subjected to 10min ischemia followed by 10min, 30min, 3h, 6h and 1d reperfusion. Bands corresponding to p-JNK were scanned and the intensities represented as values are showed in (**B**). F(11,36) = 24, ^*^ *p*<0.05 vs. sham; F(11,36) = 24, ^#^ *p*<0.05 vs. Vehicle. (**C**) Effect of E2-BSA on p-JNK at 10min of reperfusion. F(3,12) = 9, ^*^ *p*<0.05 vs. sham; F(3,12) = 9, ^#^ *p*<0.05 vs. Vehicle. (**D**) Confocal analysis of NeuN and p-JNK immunostaining in hippocampus CA1 at 10min reperfusion after global cerebral ischemia. Sham(a,b,c); vehicle(d,e,f); R10min+EDC(g,h,i); R10min+E2-BSA(j,k,l). Magnification 40× and Scale bar 50µm.

We next examined EDC and E2-BSA upon activation of JNK, a proapoptotic factor. **Fig. 4A–B** shows that p-JNK levels are increased at all times after reperfusion as compared to sham, except for the 3h time point which was not significantly elevated. Total JNK levels did not change regardless of time or treatment. EDC significantly inhibited p-JNK levels at all time points following GCI, except the 3h time point where p-JNK levels were not elevated following reperfusion. E2-BSA also significantly inhibited the elevation of p-JNK at 10min after reperfusion, while having no effect upon total JNK levels (**Fig. 4C**). Confocal analysis of NeuN and p-JNK double immunostaining confirmed the EDC and E2-BSA attenuation of p-JNK elevation at 10min reperfusion following GCI, and further demonstrated that the elevation of p-JNK occurred predominantly in CA1 neurons, although some non-NeuN positive cells also showed p-JNK staining as well (**Fig. 4D**).

#### Inhibition of ERK and PI3K-Akt Signaling Abolishes EDC and E2-BSA Neuroprotection

**Figure 5:**
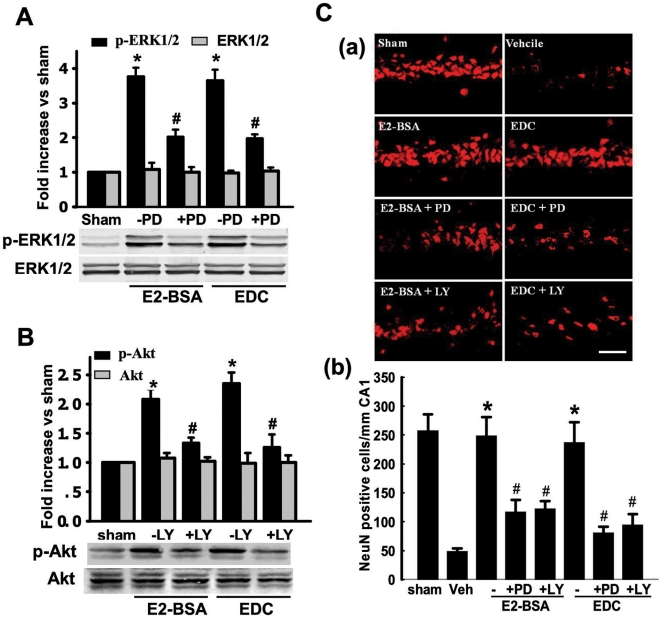
Effects of PD98059 and LY294002 on neuroprotective role by E2-BSA and EDC following cerebral ischemia in hippocampal CA1 region. (**A**) Effects of PD on p-ERK1/2 level at 10min of reperfusion in rats pre-treated with EDC or E2-BSA. F(4,15) = 121, ^*^ *p*<0.001 vs. sham; F(4,15) = 121, ^*^ *p*<0.001 vs. Vehicle. (**B**) Effects of LY on p-Akt level at 10min of reperfusion in rats pre-treated with EDC or E2-BSA. F(4,17) = 54, ^*^ *p*<0.001 vs. sham; F(4,17) = 54, ^#^ *p*<0.05 vs. Vehicle. (**C**) NeuN immunostaining showed the effects of PD or LY on surviving neurons of the hippocampal CA1 region in rats treated with EDC or E2-BSA and subjected to 10min ischemia followed by 7d reperfusion (*Fig. 5C*, *panel a*). NeuN-positive neurons in per 1mm length of CA1 region was counted as vital survival neurons as shown in Fig. 5C, panel b. F(7,32) = 68, ^*^ *p*<0.001 vs. vehicle group; F(7,32) = 68, ^#^ *p*<0.001 vs. groups pre-treated with EDC or E2-BSA. PD: PD98059, LY: LY294002, Veh: vehicle.

**Figure 6:**
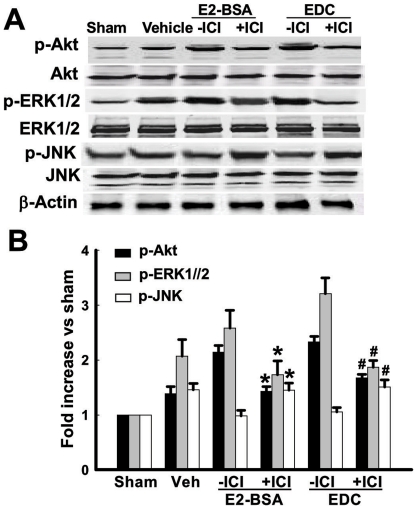
Effects of ICI 182,780 on phosphorylation of Akt, ERK1/2 and JNK induced by E2-BSA or EDC at 10min of reperfusion in hippocampal CA1 region. (**A**) Western blot analysis showed phosphorylation levels and protein expressions of Akt, ERK1/2 and JNK. Samples were obtained from sham, vehicle, E2-BSA, E2-BSA+ICI, EDC and EDC+ICI treated animals. Bands corresponding to p-Akt, p-ERK1/2 and p-JNK were scanned and the intensities represented as fold increase vs. sham as shown in (**B**). F(5,18) = 106, *p*<0.01 for p-Akt; F(5,18) = 38, *p*<0.01 for p-ERK1/2; and F(5,18) = 23 for p-JNK. ^*^ indicates vs. E2-BSA groups without ICI, ^#^ indicates vs. EDC groups without ICI. ICI: ICI 182,780.

We next examined the role of ERK and PI3K-Akt signaling in mediating EDC and E2-BSA neuroprotection following GCI. **Fig. 5A** shows that EDC and E2-BSA enhancement of p-ERK levels in the CA1 region at 10min reperfusion after GCI is abolished by pretreatment with the MEK inhibitor, PD98059. Likewise, pretreatment with the PI3K inhibitor, LY294002 markedly attenuated the ability of EDC and E2-BSA to enhance p-Akt levels in the CA1 region at 10min reperfusion after GCI (**Fig. 5B**). A functional role for ERK and Akt signaling in EDC and E2-BSA neuroprotection against GCI is suggested by the findings in **Fig. 5C (a,b)**, in which pretreatment with PD98059 or LY294002 markedly attenuated EDC and E2-BSA neuroprotection, as demonstrated by NeuN staining (**Fig. 5C,a**) and number of NeuN-positive cells (**Fig. 5C,b**) in the hippocampal CA1 region. Furthermore, as shown in **Fig. 6**, additional work suggests that EDC and E2-BSA regulatory effects upon ERK, Akt and JNK activation after GCI are mediated by estrogen receptors, as pretreatment with the ER antagonist, ICI182,780 abolished EDC and E2-BSA modulation of ERK, Akt and JNK activation in the CA1 region at 10min reperfusion.

### Activation of CREB and enhanced levels of BDNF by EDC following GCI

**Figure 7:**
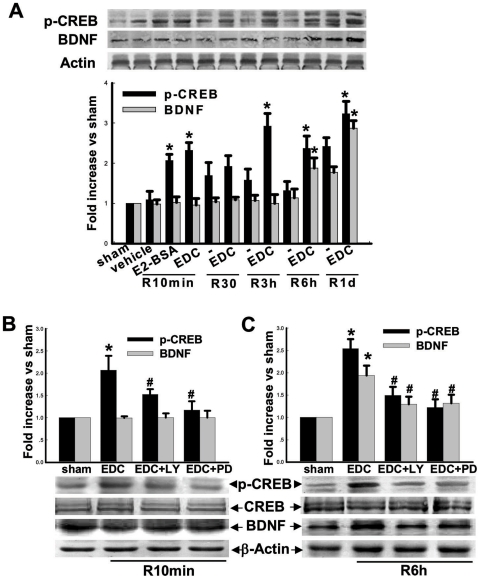
Effects of EDC on protein levels of CREB, p-CREB and BDNF following ischemic reperfusion in the hippocampal CA1 region. (**A**) Western blot analysis showed the time-courses of p-CREB, CREB and BDNF protein expression, as well as the effects of EDC or E2-BSA. F(3,16) = 31, ^*^ *p*<0.001 vs. vehicle at the same timepoint. (**B–C**) Effects of PD or LY on CREB/p-CREB level and BDNF levels at 10min of reperfusion or 6h reperfusion in rats pre-treated with EDC. B: F(3,16) = 23, ^*^ *p*<0.001 vs. sham; F(3,16) = 23, ^#^ *p*<0.001 vs. EDC group. C: F(3,12) = 64, ^*^ *p*<0.001 vs. sham; F(3,15) = 21, ^#^ *p*<0.001 vs. EDC group. R: reperfusion, PD: PD98059, LY: LY294002.

We next examined whether EDC could regulate activation of the transcription factor, CREB, and its transcriptional target BDNF in the hippocampal CA1 region after GCI. As shown in **Fig. 7A**, EDC rapidly increased pCREB levels in the CA1 region 10min post reperfusion, with a subsequent increase at 3h, 6h and 1d as compared to sham and vehicle controls. E2-BSA also increased pCREB levels similar to EDC at 10min post-reperfusion. The early elevation of pCREB levels by EDC, was followed by elevation of its transcriptional target, BDNF at latter time points (6h and 1d reperfusion) in the hippocampal CA1 region. **Fig. 7B&C** illustrates the effect of the PI3K and MEK inhibitor on EDC enhancement of pCREB and BDNF levels in the hippocampal CA1 region at 10min and 6h after reperfusion. As shown in **Fig 7B&C**, administration of the PI3K or MEK inhibitor markedly attenuated EDC-induced pCREB elevation in the CA1 region at 10min and 6h after reperfusion, as well as significantly attenuated the EDC-induced BDNF elevation at 6h after reperfusion, suggesting that PI3K-Akt and MEK-ERK signaling pathways mediate the EDC-induced elevation of pCREB and BDNF in the hippocampus following GCI.

## Discussion

The results of the current study provides evidence that activation of extranuclear estrogen receptors by E2 conjugates EDC and E2-BSA rapidly induces activation of a ERK-Akt-CREB-BDNF signaling pathway and results in neuroprotective and cognitive preserving effects in the hippocampal CA1 region following GCI. Use of fluorescent-labeled EDC and E2-BSA conjugates confirmed that the conjugates exhibit an extranuclear/membrane localization pattern, and do not enter the nucleus after *icv* injection. Some cells also showed come cytoplasmic staining/localization of the E2 conjugates. This finding agrees with previous work *in vitro* demonstrating that the E2 conjugates localize principally to the membrane with some cytoplasmic localization and are unable to enter the nucleus and activate nuclear ER, but are potent activators of rapid membrane signaling pathways [32], [33]. That the rapid signaling effects of EDC and E2-BSA observed in our study are mediated by estrogen receptors was evidenced by the fact that they were blocked by administration of the ER antagonist, ICI182,780.

Currently, it is unclear which extranuclear estrogen receptor mediates the rapid effects of E2 or the E2 conjugates used in our study. Work by Levin's group [36], [37] has shown that ERα and ERβ can exist as dimers in the plasma membrane of cells, and that COS-7 cells engineered to express ERα and ERβ display localization of ∼2–5% of ERα and ERβ protein to the plasma membrane [32]. These studies suggest that classical ERs can be targeted to the plasma membrane. Additional work demonstrated that palmitoylation of ERα and ERβ and interaction with the scaffold protein, caveolin-1, are key mechanisms for targeting ERα and ERβ to the plasma membrane [38], [39]. These studies were conducted in non-neuronal cells, however, western blot, immunohistochemical and surface biotinylated protein studies have confirmed the presence of both ERα and ERβ at the plasma membrane of neurons in the in various brain regions including the hippocampus, and at other extranuclear sites, such as in dendrites and spines [40], [41], [42], [43], [44], [45], [46]. Furthermore, membrane localization of ERα and ERβ has been demonstrated in glia cells in different brain regions [43], [45], [47], [48], and glia cells have also been implicated to participate in mediating estrogen neuroprotection via the release of growth factors and neuroactive steroids [49], [50], [51]. While in our study, we did not perform GFAP staining to determine whether astroglial cells had FITC-EDC membrane labeling, we did observe some cells in the hippocampus with typical glia morphology that displayed FITC-EDC membrane labeling. Thus, this supports the possibility that E2 may also act on glial cells to facilitate its neuroprotective protective effects, as suggested by others previously [49], [50], [51]. However, the role of glia in E2 neuroprotection requires further study as a recent study using neuron-specific and microglia-specific ERα knockout mice found that E2 neuroprotection in the cerebral cortex against focal cerebral ischemia was markedly attenuated only in the *neuron*-specific ERα knockout mice and not in the microglia-specific ERα knockout mice, suggesting that E2 may in part, act directly upon neurons to exert neuroprotection against ischemia [52]. However, the study did not examine the hippocampus, and also did not study astrocyte-specific ERα knockout mice or the role of ERβ, and thus further studies are needed to address this issue more completely.

Based on the above studies showing that both ERα and ERβ can be localized to the membrane, either extranuclear ERα or ERβ could mediate E2 rapid signaling and neuroprotective effects in the hippocampus. Use of ERα- and ERβ-selective agonists has provided evidence that activation of *either* ERα or ERβ could lead to enhanced neuroprotection following GCI [16]. However, using an antisense oligonucleotide knockdown approach, our group found that only knockdown of ERα, but not ERβ in the hippocampal CA1 region, significantly attenuated E2 rapid signaling and neuroprotective effect following GCI [15], which suggests that ERα may be more important in mediating the E2 signaling and neuroprotective effects in the hippocampus. However, we can't completely rule out a role for membrane ERβ, as the antisense knockdown only achieved a 50–60% knockdown of the ERβ protein in that study and a greater reduction may be needed to determine conclusively a role or non-role of ERβ. However, use of ER knockout mice revealed that E2 neuroprotection in focal cerebral ischemia was unaffected by complete whole animal knockout of ERβ, while, in contrast, ERα knockout mice had a dramatic loss of E2 neuroprotection [13], further supporting a critical role for ERα in E2 neuroprotective effects. Finally, it is also possible that the newly identified putative G-protein-coupled estrogen receptor, GPR30, could have a role in mediating E2 rapid signaling and neuroprotective effects [53], [54]. In support of this possibility, preliminary studies by our lab found that *icv* administration of antisense oligonucleotides to GPR30 significantly attenuated E2 rapid signaling and neuroprotection following GCI (*unpublished observations*). Thus, in addition to extranuclear ERα, extranuclear GPR30 may also participate in mediating estrogen rapid signaling and neuroprotective effects in the hippocampus. Interestingly, studies in pancreatic beta cells similarly revealed a role for *both* extranuclear ERα and GPR30 in mediating E2 protection of pancreatic beta cells from streptozotocin-induced apoptosis [55].

While our data demonstrates a potent effect of EDC (and E2-BSA) in inducing rapid *nongenomic* signaling, one should not construe that EDC only activates a nongenomic signaling pathway and is incapable of exerting genomic effects. Work by Katzenellenbogen and coworkers [56] clearly showed that EDC could regulate gene expression in cells *in vitro* and that the effect did not involve interaction with or activation of nuclear ER genomic signaling. Rather, EDC effected changes in gene expression via its activation of rapid ERK and Src kinase signaling which can regulate phosphorylation of transcription factors, histones and other factors and thereby modulate gene transcription. The study further showed that EDC was incapable of recruiting nuclear ERα to estrogen responsive regions of genes, whereas ERα recruitment by E2 was very effective. Thus, EDC nongenomic signaling can induce genomic signaling that is independent of nuclear ER. Intriguingly, previous work by Pfaff and coworkers has also demonstrated that nongenomic signaling by E2 in the hypothalamus can actually potentiate E2 genomic actions to induce lordorsis behavior [57], [58], suggesting that rapid effects of E2 may also modulate genomic effects of E2.

Interestingly, our own findings revealed that EDC and E2-BSA enhanced phosphorylation of the transcription factor, CREB in a rapid fashion following reperfusion, and that this effect is ERK- and Akt-dependent. Among the best known CREB transcriptional targets is the growth factor, BDNF, which was shown in our study to also be elevated by EDC. We also found that a second transcriptional target of CREB, Bcl-2, a potent antiapoptotic factor, was also induced by EDC in the hippocampal CA1 region following ischemic reperfusion (*unpublished observation*). These findings raise the possibility that EDC activation of extranuclear estrogen receptors may involve a nongenomic to genomic signaling cascade via kinase-induced activation of the transcription factor, CREB. It should be noted that Etgen and coworkers [59] previously demonstrated that E2 rapidly elevates pCREB in the hippocampal CA1 following GCI, while Raval et al [60] found that higher serum E2 levels on proestrus was correlated with enhanced pCREB in the hippocampus and increased neuroprotection from GCI, further supporting a potentially critical role for CREB in E2 neural actions. Our study adds to these findings by demonstrating a role for extranuclear estrogen receptors in CREB activation via the use of the nuclear impermeable E2 conjugates, demonstrating the importance of ERK and Akt signaling for CREB activation and for elevation of BDNF, and demonstrating a functional role of extranuclear estrogen receptors and the ERK-Akt-CREB-BDNF signaling pathway in preserving cognitive function following GCI.

In addition to enhancing activation of the well-known pro-survival factors, ERK, Akt and CREB, our study demonstrated that EDC exerted a prolonged attenuation of phosphorylation of JNK at *Thr183/Tyr185*, phosphorylation sites known to be critical for JNK activation [61]. JNK is known to phosphorylate many cellular proteins, including several implicated in apoptosis, and can translocate to the nucleus to activate c-Jun and AP-1-mediated gene transcription, leading to transcription of pro-death genes [62]. Previous work by our group and others has shown that administration of a JNK inhibitor [6], [63] or knockout of JNK [64] results in profound protection of the brain against cerebral ischemia, further demonstrating a key pro-apoptotic role of JNK in ischemic neuronal cell death. Thus, the ability of EDC to markedly attenuate JNK activation after GCI likely contributes significantly to its neuroprotective actions.

Finally, with respect to the cognitive preserving effects of EDC, it is suggested that EDC activation of CREB and its ability to increase BDNF in the hippocampal CA1 region likely plays a significant role in preservation of cognitive function following GCI. Both CREB and BDNF have been implicated in neuroprotection, as well as in facilitating learning and memory [21], [65], [66], [67], [68], [69]. It should be noted that the enhanced expression of BDNF by EDC was *delayed* in our study as compared to the CREB activation, as BDNF elevation occurred at 6–24h after reperfusion, while pCREB elevation occurred as early as 10min post-reperfusion. The delayed induction of BDNF could be explained by the time needed for CREB to enhance transcription of BDNF and for its translation into mature protein. The activation of CREB in the CA1 region by EDC following reperfusion was also observed at multiple time points (10min, 3h, 6h and 24h). This is intriguing and may have functional importance, as prolonged CREB activation has been correlated with enhanced neuroprotection in previous studies [65], [70]. Finally, one must consider the temporal pattern of the CREB and BDNF changes to determine the most probable mechanism of EDC preservation of cognitive function. In our study, these elevations occurred in the initial 24h time window following reperfusion, while our examination of cognitive function was 6–9 days later. Considering this, it is most likely that the role of CREB and BDNF in cognitive preservation in our study is limited to its *neuroprotective* actions that reduce ischemic neuronal cell death in the hippocampal CA1 region following GCI and thereby preserves cognitive function. However, BDNF has also been implicated to regulate synaptic plasticity [69], and such a plasticity-regulatory action in the early post-ischemic reperfusion period (first 24hr) could help maintain synaptic connections following the ischemic insult, which also could facilitate preservation of cognitive function.

In conclusion, the current study provides important new evidence that activation of extranuclear estrogen receptors with resultant induction of an ERK-Akt-CREB-BDNF signaling pathway mediates neuroprotection and preservation of cognitive function following GCI. The study thus adds to a growing literature supporting a potentially important role of extranuclear estrogen receptors in mediating estrogen beneficial effects in the brain.
